# Influence of training and competitive sessions on peripheral β-endorphin levels in training show jumping horses

**DOI:** 10.14202/vetworld.2017.67-73

**Published:** 2017-01-18

**Authors:** Cristina Cravana, P. Medica, G. Ragonese, E. Fazio

**Affiliations:** Department of Veterinary Sciences, University of Messina, Polo Universitario Annunziata, 98168 Messina, Italy

**Keywords:** β-endorphin, competition, horse, show jumping, training

## Abstract

**Aim:**

To investigate the effects of training sessions on circulating β-endorphin changes in sport horses before and after competition and to ascertain whether competition would affect this response.

**Materials and Methods:**

A total of 24 trained jumping horses were randomly assigned to one of two training groups: Group A (competing) and Group B (not competing). To determined plasma β-endorphin concentrations, two pre- and post-competition training weeks at aerobic workout and two competitive show jumping event days at anaerobic workout were measured before, 5 and 30 min after exercise. Exercise intensity is described using lactate concentrations and heart rate. The circuit design, intensity, and duration of training sessions were the same for both groups.

**Results:**

In Group A, one-way analysis of variance for repeated measures (RM-ANOVA) showed significant effects of exercise on β-endorphin changes (F=14.41; p<0.001), only in the post-competition training sessions, while in Group B showed no significant effects. Two-way RM-ANOVA showed, after post-competition training sessions, a significant difference between Group A and Group B (F=6.235; p=0.023), with higher β-endorphin changes in Group A, compared to Group B. During the competitive show jumping sessions, one-way RM ANOVA showed significant effects of exercise on β-endorphin changes (F=51.10; p<0.001). The statistical analysis, in Group A, showed a significant difference between post-competition training and competitive exercise (F=6.32; p=0.024) with higher β-endorphin values in competitive sessions compared to those of post-competition training.

**Conclusion:**

Lactate concentrations seem to be the main factors being correlated with the raise of β-endorphin during anaerobic exercise of competitive events. Exercise of low intensity, as well as that one of training sessions, does not appear to stimulate a significant increased release of β-endorphin and it may depend on the duration of the exercise program. Moreover, the responses during exercise in the course of post-competition training sessions seem to be significantly different from those the pre-competition training. These data show that the preliminary competitive stress induced additional significant changes of β-endorphin pattern. It would reflect the need of a long-lasting modulation of fatigue and pain perception related to the effect of an additional physical and mental effort for the consecutive competitive and training sessions.

## Introduction

The horse is able to improve and optimize its functional efficiency, during the execution of a given exercise, through constant training programs, aimed at preparing and optimizing the psycho-physical characteristics. Physical activity and exercise are potential conditions of psycho-physical stress for the athlete horses engaged in tasks whose severity, in terms of duration and intensity, implies different responses of the major body systems [1,2].

Changes in heart rate (HR), blood lactate and several endocrine systems, such as the hypo-pituitary-adrenal axis (HPA), are directly involved in the adaptation to exercise [3-6]. In particular, plasma levels of several hormones increase with exercise as part of the integrated response to exercise-related stress; these changes have also been used to assess performance level in horses [7-9].

It has been reported that exercise induces a marked increase in blood levels of the body’s own morphine opioids, measured as β-endorphin. This is a neuropeptide hormone known to modify the excitability of central nervous system neurons and to change pain transmission, respiration, motor activity, mood and pituitary hormone secretion. In the opioid system response to exercise-related stress, only the central effects of opioid are present. Opioid diffusion in the brain is higher than that found in peripheral blood. The opioid system is localized at the hypothalamic level and it appears that β-endorphin, completely produced by hypothalamic neurons, is co-produced with adrenocorticotropic hormone. At the SNC level, the best-known effect of β-endorphin is the analgesic one, caused by the increase in the pain threshold and by prolonged analgesic effects.

It is furthermore well-known that β-endorphin has multiple effects on mood and on several other behavioral features [10,11].

It has been suggested that the β-endorphin release could also be regulated by the adenohypophysis, or that it could depend on the adrenergic stimulation; this is, therefore, the reason why β-endorphin is considered useful, with regard to jumping horses, as a parameter in stress monitoring and in sport fitness monitoring [8,12].

The previous studies on horses revealed that circulating β-endorphin concentrations increased when intensity and duration of exercise were increased [13,14]. The time to fatigue is modulated by the rise in β-endorphin, which is also associated to impairment of performance [15]. Furthermore, in horses, such rise is considered as a marker of stress and welfare [16].

Clarification is still required with respect to the behavior of β-endorphin during competition since field data regarding the effects of exercise on β-endorphin in horses are still limited [8]. Such studies could shed some light on the effects of exercise during competition with respect to training conditions. Moreover, it is presumable that horses accustomed to exercise, compared to untrained subjects, should present lower levels of β-endorphin, lactate and HR [16-18].

In contrast, higher concentrations of β-endorphin have been observed in horses undergoing intense and fast exercise [13,14] and after jumping equestrian competition, compared to horses subjected to experimental show jumping tests [8,19].

Blood lactate analysis is commonly used by sport scientists to gain information about the metabolic response of athletes horses to various exercise intensities. Blood lactate measurements are determined during the training sessions with a hand-held portable lactate analyzer (Accusport) allowing immediate adjustments to training intensities and work prescription for individual horses. Moreover, blood lactate determination as a measure of aerobic-anaerobic threshold has been found to be useful for assessing fitness, running ability, and racing performance in equine athletes [17,20,21].

The aim of this research was to identify the trend of β-endorphin involved in maintaining endocrine homeostasis in jumping horses during pre- and post-competition training and also to ascertain whether competition would affect these responses. On this basis, it was hypothesized that the β-endorphin responses during exercise in the course of training would be significantly different from those in the jumping events days and that the competition has an effect on it.

## Materials and Methods

### Ethical approval

The study was approved by the Messina University Institutional Board for the Care and Use of Animals, and informed consent was obtained from the owners before the start of the study.

### Animals

The research was conducted on 24 trained horses (24 sella italiana; 16 geldings and 8 females; mean body weight 520±60 kg), aged from 7 to 12 years (9.3±0.7 years) which were recruited in according to their same level of show experience. All horses were in training and competing at low-medium levels. All horses were subjected to the same type of management including time and length of the daily training exercise and same groom. Subjects were randomly assigned to one of two training groups paired by gender: Group A (competing) and Group B (not competing).

Horses were all stabled at the same riding school, and each horse was kept in an individual box (4 m×4 m) with natural lighting, allowing reciprocal visual contact and with access to a sand outdoor paddock 2-3 h per day. Each horse was fed a daily ration of 8 kg of alfalfa and grass hay and 4 kg of a commercially available hay cube ration (split into two feeds). Water and a salt/mineral block were provided in the boxes *ad libitum*. The horses were considered to be clinically healthy by the referring veterinarian.

To assess the progress of the β-endorphin concentrations, all horses in the study were monitored for both two training weeks (pre- and post-competition) and for two jumping events days.

### Training sessions

#### Training sessions pre- and post-competitions

The horses’ weekly training sessions consisted of 2 consecutive days at rest, and then, 3 days of fieldwork at aerobic workout (HR was always under 150 beats/min and lactate steady states were between 3.5 and 4.1 mmol/L) with an alternation of work sessions, divided as follows:

Day 1: Flat work, lasting 50 min, with bars on the ground (10 min warm-up pace, 10 min trot in a straight line, 5 min at a gallop in a straight line, 5 min walking and 5 min at full gallop in a straight line; 10 min at the trot in a straight line, 5 min warm-down pace. During the trot and canter, the horse was made to overcome 6 fences, placed on the ground, as part of a predetermined path).

Day 2: Flat work, lasting 50 min, followed by jumping (10 min warm-up pace, with 5 min walking and 5 min trotting, 30 min trotting and jumping 6 fences, with gradually increasing heights from 65 to 120 cm; 10 min warm-down pace).

Day 3: Flat work, lasting 50 min, with bars on the ground (10 min warm-up pace, 10 min trotting in a straight line, 5 min galloping in a straight line, 5 min walking and 5 min galloping in a straight line; 10 min trotting in a straight line, 5 min warm-down pace. During the trot and gallop, all horses jumped some fences on the ground).

### Competition sessions

Over 2 consecutive days, all horses performed two competitive jumping sessions over fences of different heights: 110 cm (session 1) and 120 cm (session 2). The jumping sessions were performed after a warm-up. The warm-up consisted of a 10 min walk, trot, and quiet canter. The horses jumped two low fences before competition commenced. After the warm up, the horses walked for 10 min until the HR decreased to 50 beats/min.

Both sessions were carried out in an outdoor arena (33.77 m wide; 70.33 m long) and performed the same circuit design over 10 fences of 110 cm in the 1^st^ day and over 10 fences of 120 cm in the 2^nd^ competition’s day. Five upright and five cross-pole fences were utilized in each session. The two competitive sessions consisted of anaerobic workout (HR was always above 150 beats/min and lactate steady states were between 5.6 and 7.4 mmol/L). The horses performed the two different sessions with the same rider.

### Hormone analysis

Two venous blood samples were drawn from every horse: One, while the horse was resting in its box (07.00 AM) and the other immediately after the horse finished the exercise. Blood samples were obtained from the left jugular vein with minimal exposure to air using evacuated tubes (Venoject, Terumo; Belgium). To analyze β-endorphin concentrations, an aliquot of the blood samples (2.5 mL) was transferred after collection into polypropylene tubes containing ethylenediaminetetraacetic acid (1 mg/mL of blood) and aprotinin (500 Kallikrein Inhibitor Unit/mL blood, ICN Biomedicals Inc., Aurora, OH, USA) and kept at 4°C. Plasma samples were separated and stored at −80°C until analyzed. Peptides were extracted from plasma samples with 1% trifluoroacetic acid (high-performance liquid chromatography [HPLC] grade) and eluted with 60% acetonitrile (HPLC grade) in 1% trifluoroacetic acid.

Plasma β-endorphin concentrations were measured in duplicates, using a commercial radioimmunoassay kit (Peninsula Lab., Inc., Belmont, CA, USA) for human β-endorphin, which has a 100% cross-reactivity with equine b-endorphin [12,19]. The hormone assay used has a range for the amount of β-endorphin detected of 1-128 μL (3-371 pmol/L). The intra- and inter-assay coefficients of variation were 7% and 15%, respectively.

Blood lactate concentrations were measured using Accusport tester (Boehringer Mannheim, GmbH).

HR was monitored with a Polar Sport Tester (Polar Electro Europe BV, Fleurier, Switzerland), which recorded HR at 15 s intervals. One electrode was placed under the girth behind the left elbow, the other under the saddle at the withers. HR was recorded at 15 intervals during the exercise and until 30 min after completing the exercise. HR was adopted as a quantitative measure to assess workloads of different training and show jumping sessions.

### Statistical analysis

The statistical analysis of the parameters in the study, for both training and competition sessions, was conducted using analysis of variance for repeated measures (RM-ANOVA) to evaluate the effect of exercise over time. A two-way analysis of variance with repeated measures (two-way RM-ANOVA) was applied to test for the effects of the different type of sessions (training and competition) and sampling time, as well as for the interaction between them, on hormonal concentrations. When the F value was significant (p<0.05), the differences between individual means over time were assessed using a *post*-*hoc* multiple comparison test (Bonferroni). Correlations between the parameters in the study and between these and the fence heights were assessed by linear regression (r), calculated using Pearson’s method analysis.

## Results

### Group A (competing horses)

#### Effect of training sessions pre- and post-competition

In pre-competition training sessions, one-way RM-ANOVA showed that the exercise did not significantly affect β-endorphin changes.

In post-competition training sessions, plasma concentrations of β-endorphin showed a statistically significant increase in both the totality of the sessions (F=14.41; p<0.001) and the individual three daily sessions, respectively, in the first (F=8.30; p=0.0021), second (F=6.45; p=0.0062) and the 3^rd^ day (F=7.78; p=0.0034). As compared to basal values statistical analyses showed significant differences, with higher β-endorphin levels at 5 min (p<0.05) both in the totality of the sessions and the individual two daily training sessions. The results are presented in Table-1.

**Table-1:** Mean values (mean±SD) of β-endorphin in competing horses (Group A) after 3 consecutive days of pre- and post-competition training.

β-endorphin (pg/mL)						

Group A						

Samplings	Pre-competition			Post-competition		
	
	Basal	5 min	30 min	Basal	5 min	30 min
1^st^ day	43.66±10.78	53.43±17.08	50.34±10.07	49.15±16.91	76.42±21.08^aAA^	57.58±11.61
2^nd^ day	48.12±9.76	59.76±13.87	56.42±12.50	51.58±15.78	92.98±18.98^aA^	58.64±13.01
3^rd^ day	47.58±9.06	48.66±11.69	51.91±12.15	47.87±10.54	80.91±17.15^aA^	53.90±12.54
Total	46.45±9.78	53.95±14.16	53.22±11.50	49.53±14.44	83.44±19.07^aA^	56.71±12.49

Superscripts indicate differences versus basal values

a (p<0.05) and versus 5 min pre-competition

A (p<0.05). SD=Standard deviation

Two-way RM-ANOVA showed that the interaction first or second training weeks/time results (pre- and post-competition) were statistically significant for β-endorphin changes (F=7.56; p<0.004), with higher β-endorphin levels at 5 min (p<0.05) in post-competition sessions compared to pre-competition sessions. Over the two training weeks, there were no significant correlations between β-endorphin and lactate changes.

#### Effect of competitive sessions

**Figure-1:**
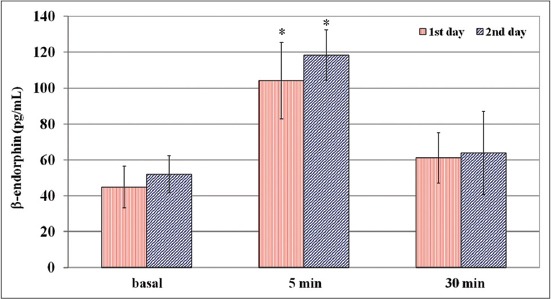
Mean values (mean±standard deviation) of β-endorphin in competing horses (Group A) after 2 consecutive days of competition. Label X: Asterisk indicates differences versus basal values (p<0.001).

In competitive sessions, plasma concentrations of β-endorphin showed a statistically significant increase both in the totality of the sessions (F=51.10; p<0.0001) and the individual two daily sessions, respectively, in the first (F=23.49; p<0.0001) and the 2^nd^ day (F=40.57; p<0.0001). As compared to basal values statistical analyses showed significant differences, with higher β-endorphin levels at 5 min (p<0.001) both in the totality of the sessions and the individual two daily competitive sessions. The results are presented in Figure-1. Two-way RM-ANOVA showed that the interaction training and competition/time results were statistically significant for β-endorphin changes (F=9.30; p=0.0059), with higher β-endorphin levels at 5 min p<0.001) in competitive sessions compared to post-competitive training sessions. There was a statistically significant positive correlation between β-endorphin and lactate changes exclusively during competitive sessions (r=0.63; p<0.001). There were no significant correlations between these parameters and the fence heights.

### Group B (not competing horses)

In all training sessions, one-way RM-ANOVA showed that the exercise did not significantly affect β-endorphin changes. The results are presented in Table-2. The interaction groups (Group A and Group B)/time results were statistically significant for β-endorphin changes (F=6.32; p=0.024), in post-competition sessions, with higher β-endorphin levels at 5 min (p<0.05) in Group A compared to Group B. There were no significant correlations between β-endorphin and lactate changes.

**Table-2:** Mean values (mean±SD) of β-endorphin in not competing horses (Group B) after 3 consecutive days of pre- and post-competition training.

β-endorphin (pg/mL)						

Group B						

Pre-competition				Post-competition		
	
Samplings	Basal	5 min	30 min	Basal	5 min	30 min
1^st^ day	42.58±15.42	53.91±10.11	47.08±14.22	48.12±7.76	57.44±17.15	51.34±10.07
2^nd^ day	44.66±10.34	59.52±11.54	49.87±10.53	40.08±11.54	60.76±13.87	53.90±11.53
3^rd^ day	49.02±12.92	58.64±21.80	52.54±15.03	47.53±14.74	54.65±16.90	48.43±15.11
Total	47.57±13.53	57.35±14.07	49.83±13.73	45.24±11.48	57.62±15.26	50.89±16.67

SD: Standard deviation

## Discussion

In all horses under this study, the hormone mean basal values were consistent with minimum values of β-endorphin at rest reported for horses, including jumpers [8,13,14,16].

The results of this study demonstrated that training sessions produced an increase of plasma β-endorphin concentrations only in Group A post-competition values, without statistically significant effects in Group B. In contrast, show jumping competition in trained horses induced significant post-exercise increases in β-endorphin concentrations with statistically significant differences between competitive and training sessions. Training exercise might be too extensive or short-lasting to induce hormone release from the anterior pituitary gland and trained jumper horses may experience higher levels of endogenous opiates more often, at least in competition.

An important stimulus for β-endorphin secretion is to exercise and its secretion is volume/intensity dependent for both aerobic and anaerobic exercise. Exercise of low intensity, as well as the training sessions in Group B, do not appear to stimulate a significant increased release of β-endorphin and may depend on duration of the exercise program. The type of exercise performed determines in large measure the effect of exercise duration on β-endorphin modifications [22]. This may imply that aside from potential differences in the threshold intensity required to stimulate a β-endorphin response with different types of exercise, the duration of low-intensity exercise may need to be prolonged to stimulate the release of β-endorphin. The lower level of intensity and shorter duration of physical exercise in training sessions, usually recorded in comparison to other sport activities [8,19], could probably justify the smallest β-endorphin response to show jumping and the lack of correlation with the lactate during the training sessions.

On the other hand, in Group A plasma concentrations of β-endorphin showed a statistically significant increase in post-competition values only, rather than for individual exercise sessions per se, as a result of the 2 consecutive days of competition. Those values were greater both than those found in the pre-competition condition and then those found in Group B. Moreover, the pre- and post-competition training exercise, in Group A, resulted in increases of plasma β-endorphin concentrations, although no correlation between β-endorphin and lactate concentrations was found.

It is possible that the extra-effort, due to the competition, is manifested by an overload of fatigue during the post-competition training, to which the body responds with release of β-endorphin greater than the corresponding post-exercise release in the pre-competition training. The higher levels of post-competition β-endorphin values in Group A compared to Group B probably reflect the need of a long-lasting β-endorphin modulation of fatigue and pain perception [15], related to the severity of competition and the time of recovery. This finding probably shows the need of increased recovery period to modulate the opioid response of β-endorphin in the post-competition training exercise. Moreover, the variability of individual pain perception might be related with significant inter-individual in the activation of the major neuroendocrine systems, like the HPA axis, in response to mental and physical stresses [23,24]. The endocrine response may represent by itself the key factor in enhancing exercise performance [25].

The increasing lactate tolerance, exercise-induced euphoria and analgesia due to β-endorphin effects are important for training in horse [26]. It would seem that β-endorphin changes could be unaffected by exercise workload by itself but may have a functional significance during post-competition training, serving as a protective measure to allow the horses to tolerate the greater workload and the emotional involvement due to competition. Moreover, the intensity during normal training sessions was much lower than during competition, suggesting that many event jumper horses are not appropriately trained.

On the other hand, excess of training may reduce β-endorphin concentrations, thus altering its beneficial effects [26], which may cause a reduced performance and a decreased workload tolerance.

Using recognized psychological and psychophysical tests, the previous studies [11] have shown that, in humans, exercise can cause euphoria, analgesia and psychological dependence similar to that experienced by the morphine addict. The degree of effort-related pain perception was lowest when the exercise-induced opioid levels were the highest. It is suggested that stress results in increased secretion from the adenohypophysis of large molecules, such as endorphins, which suppress fatigue and pain.

It may be of some significance that the horse, which has survived by outrunning its better-armed predators, has the largest pars intermedia of all domestic animals. It is possible that analgesia and motor stimulation of endogenous opioids play an important role in horse performance [7]. Although there is no direct evidence supporting this hypothesis, there is indirect evidence of the opioid influence on the exercise performance: For example, administration of naloxone, an opioid antagonist, decreases exercise performance in humans [27].

Regarding the competition results, the exercise responses in the course of the jumping event days seem to be significantly different from those of training sessions. Plasma concentrations of β-endorphin showed a statistically significant increase at 5 min post-competition values only, and returned to pre-exercise values at 30 min after the exercise. During competition, anaerobic exercise effect resulted in increases of both plasma β-endorphin concentrations and lactate with a positive correlation between β-endorphin and lactate concentrations. In this study, competitive exercise resulted in a significant increase in lactate, higher than in training session, which reflects the anaerobic metabolism of muscle cells. Similarly, it shows a muscle involvement and a high metabolic cost.

Lactate concentrations seem to be the main factor being correlated with the raise of β-endorphin during anaerobic exercise of competitive events. The lactate increase shown in the present study is quantitatively similar to what it has been previously reported with respect to racing performance in thoroughbred horses and in general in horses after competition [3,28].

These data are in agreement with previous studies, in which an effect of β-endorphin was observed (including pain perception), following changes in acid-base balance due to the presence of lactic acid [15]. In man, Schwarz and Kindermann [29] demonstrated a lactate-dependent threshold for raised β-endorphin values. Since lactate is the main agent responsible for declining pH values during high-intensity exercise, it seems tenable that its effect on the anterior pituitary gland is mediated via acidosis.

Based on the results obtained, it can be presumed that the increase in β-endorphin during competition could be the expression of both the following change in acid-base balance and the emotional involvement of the horses, in agreement with the results previously reported in the literature after competitive exercise [30] and non-competitive exercise [8,19], sub-and supra-maximal [31-33]. During incremental exercise tests, plasma β-endorphin concentrations were positively correlated with both exercise speed [31] and intensity, with a critical threshold for β-endorphin release [4,13,14].

Moreover, the absence of a significant correlation of β-endorphin with fence heights confirms data previously obtained in both jumping horses after competition [8] and experimental tests of equestrian competition [34].

## Conclusions

The results suggest, therefore, that the endogenous opioid system modulates the stress response to exercise, ensuring its action even after the pre- and post-competition training sessions with significant differences from those found in the jumping event days. Probably, these findings show the need of increased recovery period to modulate the opioid response of β-endorphin in the post-competition training exercise following the subjective stress perception during emotional conditions and the greater workload related to the severity of competition.

## Authors’ Contributions

All authors have made substantial contributions to each step of experimental procedure and manuscript preparation. In particular: The idea for the paper was conceived by EF and PM. The experiments were performed by GR and CC. The data were analyzed CC. The paper was written by CC and PM. All authors have read and approved the final manuscript.

## References

[1] Medica P, Cravana C, Fazio E, Ferlazzo A (2011). Hormonal responses of quarter horses to a 6-week conventional western-riding training programme. Livest. Sci.

[2] Bartolomé E, Cockram M.S (2016). Potential effects of stress on the performance of sports horses. J. Equine Vet. Sci.

[3] Evans D.L, Harris R.C, Snow D.H (1993). Correlation of the racing performance with blood lactate and heart rate after exercise in thoroughbred horses. Equine Vet. J.

[4] Art T, Franchimont P, Lekeux P (1994). Plasma β-endorphin response of thoroughbred horses to maximal exercise. Vet. Rec.

[5] Beneke R, Leithauser R.M, Ochentel O (2011). Blood lactate diagnostics in exercise testing and training. Int. J. Sports Physiol. Perform.

[6] von Lewinski M, Biau S, Erber R, Ille N, Aurich J, Faure J.M, Möstl E, Aurich C (2013). Cortisol release, heart rate and heart rate variability in the horse and its rider: Different responses to training and performance. Vet. J.

[7] Golynski M, Krumrych W, Lutnicki K (2011). The role of beta-endorphin in horses: A review. Vet. Med.

[8] Ferlazzo A, Medica P, Cravana C, Fazio E (2012). Circulating b-endorphin, adrenocorticotropin, and cortisol concentrations of horses before and after competitive show jumping with different fence heights. J. Equine Vet. Sci.

[9] Ferlazzo A, Fazio E, Cravana C, Medica P (2014). Changes of circulating total and free iodothyronines in horses after competitive show jumping with different fence height. J. Equine Vet. Sci.

[10] Veening J.G, Barendregt H.P (2015). The effects of beta-endorphin: State change modification. Fluids Barriers. CNS.

[11] Bodnar R.J (2016). Endogenous opiates and behavior: 2014. Peptides.

[12] Canali E, Ferrante V, Mattiello S, Sacerdote P, Panerai A.E, Lebelt D (1996). Plasma levels of β-endorphin and *in vitro* lymphocyte proliferation as indicators of welfare in horses in normal or restrained conditions. Pferdeheilkunde.

[13] Mehl M.L, Sarkar D.K, Schott H C, Brown J.A, Sampson S.N, Bayly W.M (1999). Equine plasma beta-endorphin concentrations are affected by exercise intensity and time of day. Equine Vet. J. Suppl.

[14] Mehl M.L, Schott H C, Sarkar D.K, Bayly W.M (2000). Effects of exercise intensity and duration on plasma b-endorphin concentrations in horses. Am. J. Vet. Res.

[15] Adler G.K (2000). Exercise and fatigue-is neuroendocrinology an important factor?. J. Clin. Endocrinol. Metab.

[16] Hydbring E, Nyman S, Dahlborn K (1996). Changes in plasma cortisol, plasma β-endorphin, heart rate, haematocrit and plasma protein concentration in horses during restraint and use of a naso-gastric tube. Pferdeheilkunde.

[17] Lindner A, Fazio E, Medica P, Ferlazzo A (2002). Effect of age, time record and V_4_ on plasma cortisol concentration in standardbred racehorses during exercise. Pferdeheilkunde.

[18] Lindner A, Mosen H, Kissenbeck S, Fuhrmann H, Sallmann H.P (2009). Effect of blood lactate-guided conditioning of horses of differing durations and intensities on heart rate and biochemical blood variables. J. Anim. Sci.

[19] Cravana C, Medica P, Prestopino M, Fazio E, Ferlazzo A (2010). Effects of competitive and noncompetitive show jumping on total and free iodothyronines, β-endorphin, ACTH and cortisol levels of horses. Equine Vet. J. Suppl.

[20] Fraipont A, Van Erck E, Ramery E, Fortier G, Lekeux P, Art T (2012). Assessing fitness in endurance horses. Can. Vet. J.

[21] Lindner A, Fazio E, Ferlazzo A.M, Medica P, Ferlazzo A (2000). Plasma cortisol concentration in thoroughbred horses during and after standardized exercise tests on a treadmill and effects of conditioning on basal cortisol values. Pferdeheilkunde.

[22] Goldfarb A.H, Hatfield B.D, Armstrong D, Potts J (1990). Plasma beta endorphin concentration: Response to intensity and duration of exercise. Med. Sci. Sports Exerc.

[23] Cockrem J.F (2013). Individual variation in glucocorticoid stress responses in animal. Gen. Comp. Endocrinol.

[24] Stefánsdóttir G.I, Ragnarsson S, Gunnarsson V, Jansson A (2014). Physiological response to a breed evaluation field test in Icelandic horses. Animal.

[25] Viru M, Hackney A.C, Karelson K, Janson T, Kuus M, Viru A (2010). Competition effects on physiological response to exercise: Performance, cardiorespiratory and hormonal factors. Acta Physiol. Hung.

[26] Cuhna G.S, Ribeiro J.L, Oliveira A.R (2008). Levels of beta-endorphin in response to exercise and overtraining. Arq. Bras. Endocrinol. Metab.

[27] Mercadante S (2015). Opioid metabolism and clinical aspects. Eur. J. Pharmacol.

[28] Serrano M.G, Evans D.L, Hodgson J.L (2002). Heart rate and blood lactate responses during exercise in preparation for evening competition. Equine Vet. J. Suppl.

[29] Schwarz L, Kindermann W (1990). Beta-endorphin, adrenocorticotropic hormone, cortisol and catecholamines during aerobic and anaerobic exercise. Eur. J. Appl. Occup. Physiol.

[30] Malinowski K, Shock E.J, Rochelle P, Kearns C.F, Guirnalda P.D, McKeever K.H (2006). Plasma β-endorphin, cortisol and immune responses to acute exercise are altered by age and exercise training in horses. Equine Vet. J. Suppl.

[31] McCarthy R.N, Jeffcott L.B, Funder J.W, Fullerton M, Clarke I.J (1991). Plasma beta-endorphin and adrenocorticotrophin in young horses in training. Aust. Vet. J.

[32] McCarthy R.N, Jeffcott B, Clarke I.J (1993). Preliminary studies on the use of plasma b-endorphin in horses as an indicator of stress and pain. J. Equine Vet. Sci.

[33] Golland L.C, Evans D.L, Stone G.M, Tyler-McGowan C.M, Hodgson D.R, Rose R.J (1999). Plasma cortisol and beta-endorphin concentrations in trained and over-trained racehorses. Pflugers Arch.

[34] Ferlazzo A, Medica P, Cravana C, Fazio E (2009). Endocrine changes after experimental showjumping. Comp. Exerc. Physiol.

